# Early hyperoxemia is associated with lower adjusted mortality after severe trauma: results from a French registry

**DOI:** 10.1186/s13054-020-03274-x

**Published:** 2020-10-12

**Authors:** Josefine S. Baekgaard, Paer-Selim Abback, Marouane Boubaya, Jean-Denis Moyer, Delphine Garrigue, Mathieu Raux, Benoit Champigneulle, Guillaume Dubreuil, Julien Pottecher, Philippe Laitselart, Fleur Laloum, Coralie Bloch-Queyrat, Frédéric Adnet, Catherine Paugam-Burtz, Traumabase® Study Group, Romain Pirracchio, Anne Godier, Anatole Harrois, Thomas Geeraerts, Eric Meaudre, Sylvain Ausset, Tobias Gauss, Alain Meyer, Sophie Hamada, Arthur Neuschwander, Fabrice Cook, Helene Vinour, Jean Luc Hanouz, Arnaud Foucrier, Mathieu Boutonnet, Pascal Raclot, James Arthur, Nathalie Bruneau, Jean Cotte, Marc Leone, Gerard Audibert

**Affiliations:** 1 Urgences et Samu 93, AP-HP, Avicenne Hospital, Inserm U942, 93000 Bobigny, France; 2 Department of Anesthesia, Section 4231, Centre of Head and Orthopedics, Rigshospitalet, University of Copenhagen, Juliane Maries Vej 10, DK-2100 Copenhagen, Denmark; 3 Department of Anesthesia and Critical Care, Beaujon Hospital, AP-HP, University of Paris, Paris, France; 4 URC CRC, Avicenne Hospital, Bobigny, France; 5 Department of Anesthesia and Critical Care, CHU de Lille, Lille, France; 6 Sorbonne Université, INSERM, UMRS1158 Neurophysiologie Respiratoire Expérimentale et Clinique; AP-HP Groupe Hospitalier Universitaire APHP-Sorbonne Université, site Pitié-Salpêtrière, Département d’Anesthésie Réanimation, F-75013 Paris, France; 7 Surgical Intensive Care Unit, Georges Pompidou European Hospital, AP-HP, Paris, France; 8 Department of Anesthesia and Critical Care, AP-HP, Bicêtre Hospital, Paris, France; 9 Department of Anesthesia and Surgical Critical Care, Strasbourg University Hospital, Strasbourg, France; 10 Department of Anesthesia, Percy Army Training Hospital, Paris, France; 11 Department of Anesthesia and Critical Care, University Hospital of Reims, Reims, France; 12 Service d’Anesthésie-réanimation, Hôpital Européen Georges Pompidou, Université Paris Descartes, Paris, France; 13 Université Paris Sud, Université Paris Saclay, Department of Anesthesiology and Critical Care, Assistance Publique-Hôpitaux de Paris (AP-HP), Bicêtre Hôpitaux Universitaires Paris Sud, 78 rue du Général Leclerc, 94275 Le Kremlin Bicêtre, F-94275 Le Kremlin Bicêtre, France; 14 Anesthesiology and Critical Care Department, University Hospital of Toulouse, University Toulouse 3-Paul Sabatier, Toulouse, France; 15 Department of Anesthesiology and Intensive Care, Military Hospital, Hôpital d’Instruction des Armées Sainte-Anne, Toulon, France; 16 Emergency department, Military Hospital, Hôpital d’Instruction des Armées Sainte-Anne, Toulon, France; 17 Department of Anesthesiology and Critical Care, Percy military hospital, Clamart, France; 18 Unités de Réanimation Chirurgicale et de Surveillance Continue, Hôpitaux Universitaires de Strasbourg, Strasbourg, France; 19 Department of Anaesthesia and Intensive Care Medicine, Henri Mondor University Hospital, Creteil, France; 20 Anaesthesiology and Critical Care Department, University Hospital of Toulouse, University Toulouse 3-Paul Sabatier, Toulouse, France; 21 Department of Anesthesiology and Critical Care Medicine, Pôle Réanimations Anesthésie SAMU, Caen University Hospital, Caen, France; 22 Reims University Hospital, Robert Debré Hospital, Intensive Care Unit, Reims, France; 23 Sorbonne Université and Department of Anesthesiology and Critical Care, AP-HP, Hôpitaux Universitaires Pitié-Salpêtrière, Paris, France; 24 Pôle d’Anesthésie-Réanimation, CHU de Lille, Lille, France; 25 Department of Anesthesiology and Intensive Care Medicine, Aix-Marseille University, Assistance Publique Hôpitaux de Marseille, Hôpital Nord, Marseille, France; 26 Department of Anesthesiology and surgical Intensive Care, University Hospital of Nancy, Nancy, France

**Keywords:** Hyperoxemia, Hyperoxia, Trauma, Critical care, Oxygen

## Abstract

**Background:**

Hyperoxemia has been associated with increased mortality in critically ill patients, but little is known about its effect in trauma patients. The objective of this study was to assess the association between early hyperoxemia and in-hospital mortality after severe trauma. We hypothesized that a PaO_2_ ≥ 150 mmHg on admission was associated with increased in-hospital mortality.

**Methods:**

Using data issued from a multicenter prospective trauma registry in France, we included trauma patients managed by the emergency medical services between May 2016 and March 2019 and admitted to a level I trauma center. Early hyperoxemia was defined as an arterial oxygen tension (PaO_2_) above 150 mmHg measured on hospital admission. In-hospital mortality was compared between normoxemic (150 > PaO_2_ ≥ 60 mmHg) and hyperoxemic patients using a propensity-score model with predetermined variables (gender, age, prehospital heart rate and systolic blood pressure, temperature, hemoglobin and arterial lactate, use of mechanical ventilation, presence of traumatic brain injury (TBI), initial Glasgow Coma Scale score, Injury Severity Score (ISS), American Society of Anesthesiologists physical health class > I, and presence of hemorrhagic shock).

**Results:**

A total of 5912 patients were analyzed. The median age was 39 [26–55] years and 78% were male. More than half (53%) of the patients had an ISS above 15, and 32% had traumatic brain injury. On univariate analysis, the in-hospital mortality was higher in hyperoxemic patients compared to normoxemic patients (12% versus 9%, *p* < 0.0001). However, after propensity score matching, we found a significantly lower in-hospital mortality in hyperoxemic patients compared to normoxemic patients (OR 0.59 [0.50–0.70], *p* < 0.0001).

**Conclusion:**

In this large observational study, early hyperoxemia in trauma patients was associated with reduced adjusted in-hospital mortality. This result contrasts the unadjusted in-hospital mortality as well as numerous other findings reported in acutely and critically ill patients. The study calls for a randomized clinical trial to further investigate this association.

## Introduction

Each year, 5.8 million people die as result of trauma making it the leading cause of death for individuals below 45 years of age [1]. Furthermore, trauma constitutes a major economic burden, as trauma-related costs were estimated to $671 billion in 2013 in the USA alone [2]. Efforts to lower the mortality and morbidity following trauma are therefore of highest importance. The prehospital management of severe trauma patients requires a rapid approach during which it is recommended to provide supplemental oxygen to both treat and prevent hypoxemia [3, 4]. As a result, high fractions of inspired oxygen (FiO_2_) are commonly administered during this initial phase and may result in hyperoxemia on hospital admission. However, exposure to high oxygen levels, even during a short period of time, has been associated with cerebral and coronary vasoconstriction, deleterious effects on lung function, and increased production of reactive oxygen species [5–10].

In a large meta-analysis on randomized controlled trials (RCT), which compared liberal and conservative oxygenation administration in acutely ill patients, the relative risk of in-hospital mortality was increased amongst patients treated with a liberal oxygen approach compared to a conservative oxygen approach [11]. A recent systematic review also investigated the relationship between hyperoxemia and mortality in critically ill patients and found a similar association [12].

Despite an increasing awareness of the potentially deleterious effects of elevated arterial oxygen partial pressure (PaO_2_) in acutely ill patients [13], the prevalence of hyperoxemia in the emergency department (ED) and the intensive care unit (ICU) remains high [14–16]. Furthermore, a recent cohort study found a link between early hyperoxemia in the ED and mortality [14].

However, the association between hyperoxemia and mortality in the trauma population remains controversial. In one RCT, authors found no effect of exposure to different levels of FiO_2_ on mortality amongst patients suffering from traumatic brain injury (TBI) [17]. A recent observational study on 24,148 mechanically ventilated patients with TBI found no effects of hyperoxemia on mortality either [18].

Taken as a whole, knowledge on the effects of hyperoxemia in trauma patients is sparse and the evidence for systematic oxygen therapy in these patients is thus inadequate, especially in the pre-hospital setting [19].

The primary objective of this study was to assess the association between elevated PaO_2_ on hospital admission and in-hospital mortality in level I trauma centers. We hypothesized that a PaO_2_ ≥ 150 mmHg on admission was associated with increased in-hospital mortality.

## Methods

### Study design

This was an observational study using a multicenter, prospective trauma registry in France, the TraumaBase©. The TraumaBase consecutively collects data on trauma patients from 15 trauma centers in France. A central administrator monitors the data and the TraumaBase is approved by the Institutional Review Board as well as the National Commission on Informatics and Liberties. The study is reported in accordance with the STROBE guidelines [20].

### Setting

Between May 2016 and March 2019, data collected from the 14 level 1 trauma centers was reviewed (one center had not yet included patients). As previously described [21], the French EMS system consists of two levels of triage that will trigger a paramedic-staffed ambulance or a physician-staffed mobile ICU (*Service Mobile d’Urgence et de Réanimation* (SMUR)). In case of major trauma, the SMUR will always be activated and accompany the patient to a specialized trauma center.

### Participants

Trauma patients above 17 years of age with a PaO_2_ measured and registered in the TraumaBase® registry were included. Hypoxemic patients (PaO_2_ < 60 mmHg on arrival) and patients withdrawn from life-sustaining therapy were excluded. Baseline characteristics on hypoxemic patients can be found in the Additional file 1*.*

### Variables

The following variables were extracted from the database: age (years), gender, American Society of Anesthesiologists (ASA) score, initial Glasgow Coma Scale (GCS) score, pre-hospital systolic blood pressure and heart rate, mechanism and site of injury, volume fluid replacement (mL of colloids and/or crystalloids), catecholamine administration, use of mechanical ventilation, body temperature, arterial blood gas analysis on admission, lactate level, hemoglobin level, creatinine level, presence of hemorrhagic shock (defined as at the transfusion of at least four units of packed red blood cells within 6 h), TBI (at least one visible lesion on computed tomography), Injury Severity Score (ISS), in-hospital length of stay, and in-hospital mortality.

### Statistical methods

Patients were divided into two groups of exposure a priori according to their initial PaO_2_ on hospital admission: normoxemia (PaO_2_ 60–150 mmHg) and hyperoxemia (PaO_2_ ≥ 150 mmHg). The 150 mmHg cut-off was used as this has previously been done in an RCT on ICU patients [22], as well as in several observational studies [23–25].

Our primary aim was to assess the correlation between hyperoxemia on hospital admission and in-hospital mortality. Two pre-planned subgroup analyses on patients with an initial GCS < 8 and mechanically ventilated patients were also planned.

Categorical variables are expressed as numbers with percentages (%) and continuous variables as means with standard deviations (SD), or medians with interquartile ranges [IQR]. Characteristics were compared using a chi^2^ test for categorical data and *t* test or Mann-Whitney *U* test for continuous data.

Since hyperoxemia is caused by exposure to high oxygen levels, the association between hyperoxemia and in-hospital mortality was assessed using propensity score to reduce potential selection bias due to measured baseline covaries. The variables included in the model were chosen a priori by comparing pre-hospital variables and baseline characteristics between patients that died and survived to hospital discharge. Significant determinants of mortality were included.

The score was estimated using logistic regression, and the primary analyses were made using inverse probability of treatment weighting (IPTW).

To verify the robustness of the results, two sensitivity analyses were performed using a propensity score analysis with a matching method with a 1:1 ratio within a caliper of 0.05 standard deviation of the logit propensity score and a stratification on the quintiles of the propensity score. To account for missing data, analyses were conducted using multiple imputations by chained equations with 10 imputations obtained after 10 iterations [26]. A complete-case analysis was also performed to verify the results. The propensity scores came from 10 independent complete data sets and were averaged according to the “across approach” [27]. Balance in potentials confounders were assessed by standardized mean differences which came from a complete imputed data set [28]. A multivariate full model including factors used in the propensity score was also performed to verify the results of the propensity score.

Finally, several sensitivity analyses were performed. An analysis removing patients who died within 24 h of hospital admission was carried out to allow sufficient time for deleterious effects such as lung complications of oxygen to develop, and an analysis on patients with a GCS < 8 as well as an analysis on intubated patients was done. Furthermore, other cutoffs for hyperoxemia were examined (PaO_2_ ≥ 100 mmHg and PaO_2_ ≥ 200 mmHg), and the PaO_2_/FiO_2_ was explored using the Berlin definition [29].

All tests were two-tailed, and the results were considered statistically significant when *p* < 0.05. Analyses were performed using R statistical software [30].

## Results

**Fig. 1:**
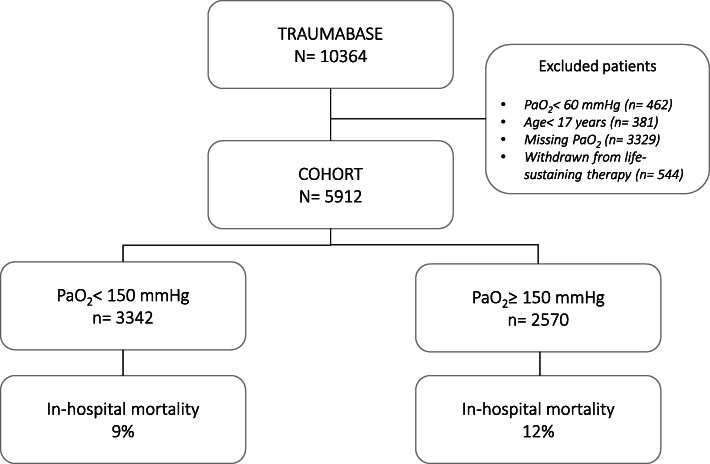
Flowchart of the included trauma patients

Of 6654 adult trauma patients with PaO_2_ values available in the database, 544 were excluded as they were withdrawn from life-sustaining therapy and 462 were excluded as they were hypoxemic on arrival, leaving 5912 patients for analysis (Fig. 1).

The median age was 39 years and the majority were males (Table 1). More than half of all patients had an ISS score above 15, and one third presented with TBI. The overall in-hospital mortality was 10%.

**Table 1:** Baseline characteristics of all included trauma patients including a comparison of norm- and hyperoxemic patients. Results are presented as medians with [interquartile ranges], numbers with (percentages), or as otherwise indicated

	All patients	Normoxemic 60 < PaO_2_ < 150 mmHg	Hyperoxemic PaO_2_ ≥ 150 mmHg	*p* value
	*N* = 5912	*n* = 3342	*n* = 2570	
Age	39 [26–55]	41 [17–96]	36 [17–96]	< 0.0001
Sex (female)	1273 (21.6)	703 (21.1)	570 (22.3)	0.3
ASA-score > 1	1903 (34.5)	1168 (37.0)	735 (31.1)	< 0.0001
Mechanism of injury				
Falls from height	1368 (21.5)	747 (22.4)	521 (20.3)	0.089
Falls from standing	240 (4.1)	132 (4.0)	108 (4.2)	
Vehicle incident/collision	3339 (56.5)	1895 (56.7)	1444 (56.2)	
Shootings	590 (10.0)	211 (9.3)	279 (10.9)	
Fight	204 (3.5)	117 (3.5)	87 (3.4)	
Other	270 (4.6)	139 (4.2)	131 (5.1)	
Site of injury				
Head and neck	2823 (51.1)	1461 (46.9)	1362 (64.4)	< 0.0001
Face	1389 (25.1)	707 (22.7)	682 (28.3)	< 0.0001
Abdomen	1833 (33.2)	1022 (32.8)	811 (33.6)	0.56
Chest	2865 (51.8)	1647 (52.9)	1218 (50.5)	0.079
External	924 (16.7)	533 (17.1)	391 (16.2)	0.39
Extremities	3080 (55.7)	1684 (54.1)	1396 (57.9)	0.0055
Duration of prehospital care (minutes), median [IQR]	70 [48–100]	79 [49–97]	70 [45–105]	0.58
Prehospital systolic blood pressure (mmHg)	127 [110–141]	130 [0–256]	124 [0–230]	< 0.0001
Prehospital heart rate (bpm)	89 [75–105]	88 [0–170]	76 [0–155]	< 0.0001
Prehospital intubation	1840 (31.7)	651 (19.9)	1189 (47.0)	< 0.0001
Prehospital GCS score	15 [11–15]	15 [3–15]	14 [3–15]	< 0.0001
Values on hospital arrival				
pH	7.4 [7.3–7.4]	7.4 [7.3–7.4]	7.3 [7.3–7.4]	< 0.0001
PaO_2_	133 [93–216]	97 [81–117]	230 [186–308]	–
PCO_2_	40 [35–44]	39 [35–43]	40 [26–45]	< 0.0001
Temperature (°C)	36.5 [35.9–37.0]	36.6 [26.4–41.0]	36.4 [26.0–40]	< 0.0001
Lactate (mmol/L)	1.9 [1.2–3.0]	1.8 [0.2–23.4]	2 [0.2–25]	< 0.0001
Creatinine (μmol/L)	77 [65–92]	77 [8–1004]	77 [7–926]	0.64
Hemoglobin (mmol/L)	13 [11.5–14.2]	13.3 [3.9–21.6]	12.6 [1.1–20.0]	< 0.0001
Catecholamine administration	815 (14.3)	322 (10.0)	493 (19.9)	< 0.0001
Fluid replacement	500 [250–1000]	500 [0–7000]	750 [0–5500]	< 0.0001
ISS score	16 [9–25]	13 [8–24]	18 [10–27]	< 0.0001
ISS score > 15	2935 (52.9)	1433 (45.9)	1502 (62.0)	< 0.0001
Traumatic brain injury	1836 (31.6)	824 (25.1)	1012 (40.1)	< 0.0001
Hemorrhagic shock	545 (9.4)	202 (6.2)	343 (13.6)	< 0.0001
In-hospital mortality^a^	481 (10.0)	239 (8.7)	242 (11.6)	< 0.0001
Cause of death (available for 426 patients)				< 0.01
Hemorrhagic shock	46 (10.8)	21 (10.0)	25 (11.5)	
Septic chock	6 (1.4)	3 (1.4)	3 (1.4)	
Multi organ failure	98 (23.0)	59 (28.2)	39 (18.0)	
Brain death	197 (46.2)	85 (40.7)	112 (51.6)	
Traumatic brian injury	58 (13.6)	26 (12.4)	32 (14.7)	
Other	21 (4.9)	15 (7.1)	6 (2.8)	

The provided pre-hospital vital signs are the first vital signs recorded on-scene

*Abbreviations*: *ASA*, American Society of Anesthesiologists; *GCS*, Glasgow Coma Scale score; *ISS*, Injury Severity Score; *Hemorrhagic shock*, defined as administration of at least four units of packed red blood cells within 6 h; *Fluid replacement*, mL of colloids and/or crystalloids

^a^Missing in 18%. Imputated in the propensity score analysis

On hospital admission, the median PaO_2_ of the entire cohort was 133 mmHg: 3342 (57%) were normoxemic, and 2570 (43%) were hyperoxemic. Numerous baseline characteristics were significantly different between normoxemic and hyperoxemic patients: a higher proportion of hyperoxemic patients were mechanically ventilated (a comparison of baseline characteristics between intubated and spontaneously breathing patients can be found in Additional file 2), they had lower prehospital GCS scores and more suffered from a TBI. On univariate analysis, the in-hospital mortality was higher for hyperoxemic patients (12% versus 9%, *p* < 0.0001) (Table 1).

**Fig. 2:**
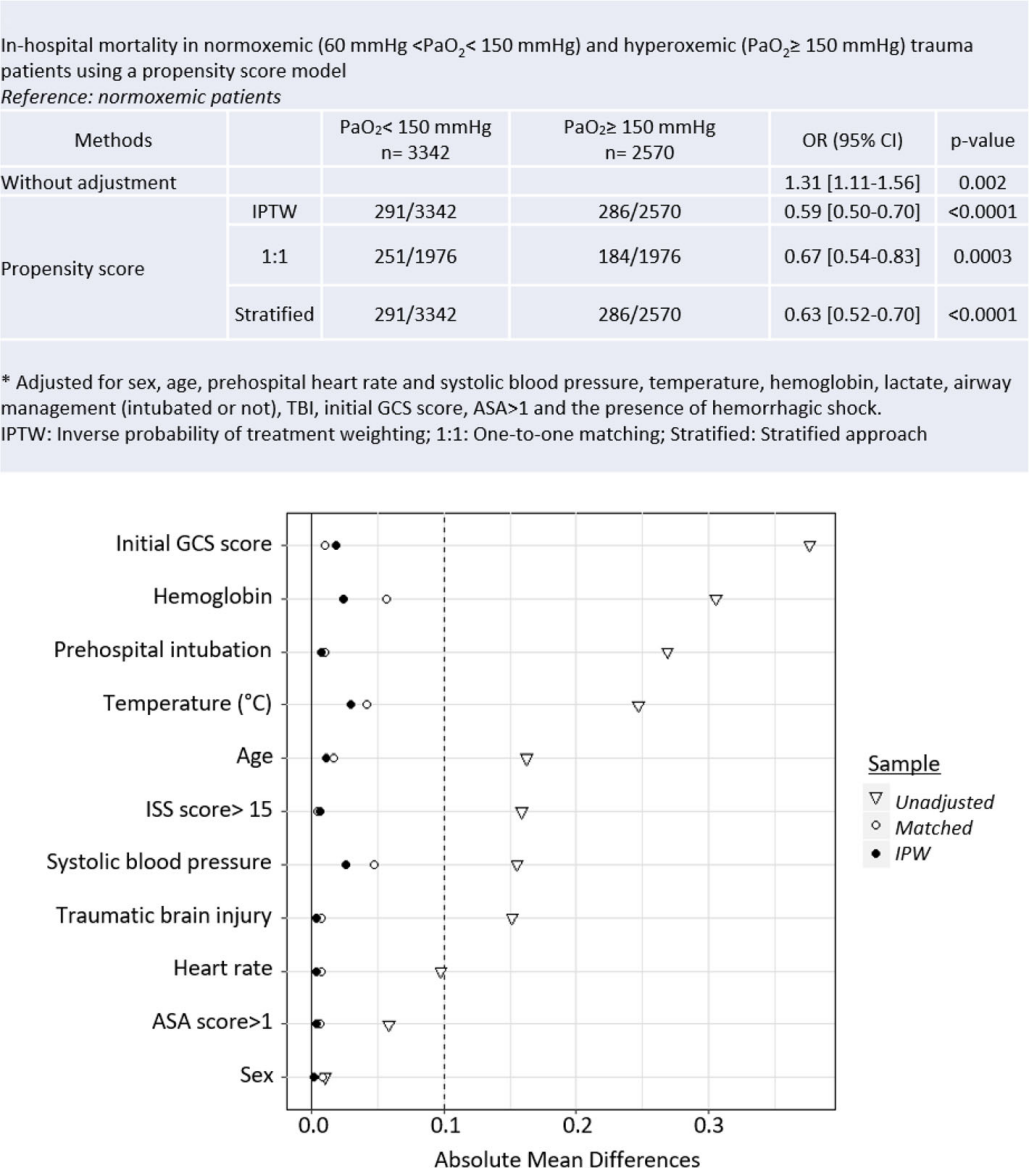
In-hospital mortality in normoxemic (60 mmHg <PaO_2_ < 150 mmHg) and hyperoxemic (PaO_2_ > 150 mmHg) trauma patients using a propensity score model

In a propensity score model, patients were matched based upon significant determinants of mortality amongst the baseline characteristics (Table 2). The model revealed an inverse relationship between hyperoxemia and in-hospital mortality: mortality was significantly decreased in hyperoxemic patients compared to normoxemic patients (OR 0.59 [0.50–0.70], *p* < 0.0001) and hyperoxemia thus appeared as a protective factor. The accuracy of the model is presented in Fig. 2. Here, the balances in potentials confounders were also checked, and the absolute mean differences were all less than 5% after using propensity score (IPTW and matching methods). The multivariate full model including factors used in propensity score verified the results of the propensity score (Additional file 3). A complete-case analysis presented very similar results (OR 0.60 [0.46–0.78], *p* < 0.0001).

**Table 2:** Baseline differences amongst trauma patients that survived to hospital-discharge or died in-hospital. Results are presented as medians with [interquartile ranges], numbers with (percentages), or as otherwise indicated

	In-hospital mortality		*p* value
	*Survived*	*Deceased*	
Age	37 [17–96]	53 [17–96]	< 0.0001
Sex (female)	906 (20.9)	123 (25.6)	0.019
ASA-score > 1	1328 (32.2)	223 (32.8)	< 0.0001
Prehospital systolic blood pressure (mmHg)	128 [0–237]	129 [0–237]	< 0.0001
Prehospital heart rate (bpm)	90 [0–240]	85 [0–200]	0.005
Prehospital intubation	1190 (27.8)	365 (77.3)	< 0.0001
Prehospital GCS score	15 [3–15]	4 [3–15]	< 0.0001
Values on hospital arrival			
PaO_2_	131 [60–812]	151 [60–609]	0.011
PaO_2_ ≥ 150 mmHg	1842 (42.4)	242 (50.3)	0.001
Temperature (°C)	36.5 [26.4–40.5]	35.5 [30.0–41.0]	< 0.0001
Lactate (mmol/L)	1.9 [0.2–24]	3.5 [0.4–24]	< 0.0001
Creatinine (μmol/L)	76 (7–1001]	94 [29–950]	< 0.0001
Hemoglobin (mmol/L)	13.1 [1.1–21.6]	11.4 [1.8–19]	< 0.0001
Catecholamine administration	461 (10.9)	223 (48.9)	< 0.0001
Fluid replacement	500 [0–6500]	1000 [0–5500]	< 0.0001
ISS score	14 [9–24]	29 [25–41]	< 0.0001
ISS score > 15	2024 (48.7)	417 (90.7)	< 0.0001
Traumatic brain injury	1209 (28.0)	329 (68.7)	< 0.0001
Hemorrhagic shock	320 (7.4)	148 (30.8)	< 0.0001

*Abbreviations*: *ASA*, American Society of Anesthesiologists; *GCS*, Glasgow Coma Scale score; *Hemorrhagic shock*, defined as administration of at least four units of packed red blood cells within 6 h; *Fluid replacement*, mL of colloids and/or crystalloids

In a sensitivity analysis, where patients deceased within 24 h were excluded, the results remained statistically significant (OR 0.63 [0.52–0.76], *p* < 0.0001) (Table 3).

**Table 3:** Sensitivity analyses. In-hospital mortality amongst subgroups of trauma patients (*reference: Normoxemia)*

**Subgroup**			
Survival beyond 24 h	Propensity score (IPTW)	0.63 [0.52–0.75]	< 0.0001
GCS < 8	Without adjustment	0.55 [0.43–0.71]	< 0.0001
	Propensity score (IPTW)	0.69 [0.53–0.89]	0.005
Mechanically ventilated patients	Without adjustment	0.52 [0.42–0.65]	< 0.0001
	Propensity score (IPTW)	0.62 [0.50–0.77]	< 0.0001
**Cutoffs for hyperoxemia**			
PaO_2_/FiO_2_ ≥ 300	Without adjustment	0.32 [0.27–0.38]	< 0.0001
	Propensity score (IPTW)	0.74 [0.62–0.88]	0.0007
PaO_2_ ≥ 100 mmHg	Without adjustment	1.03 [0.86–1.25]	0.73
	Propensity score (IPTW)	0.54 [0.46–0.64]	< 0.0001
PaO_2_ ≥ 200 mmHg	Without adjustment	1.38 [1.15–1.65]	0.0005
	Propensity score (IPTW)	0.72 [0.59–0.87]	0.0006

*GCS* Glasgow Coma Scale, *IPTW* inverse probability of treatment weighting

Likewise, in our subgroup analysis on patients with a GCS < 8, mortality was also decreased in hyperoxemic patients (OR 0.69 [0.53–0.89], *p* = 0.005). The same was true in a subgroup analysis on intubated patients (OR 0.62 [0.50–0.77], *p* < 0.0001) (Table 3).

Furthermore, our sensitivity analyses also showed a beneficial effect on mortality using PaO_2_/FiO_2_ ≥ 300, and different cut-off levels for hyperoxemia (PaO_2_ ≥ 100 mmHg and PaO_2_ ≥ 200 mmHg) left our results largely unaltered (Table 3).

## Discussion

In this large observational study of nearly 6000 trauma patients, we found hyperoxemia above 150 mmHg on hospital admission to be independently associated with a significantly decreased in-hospital mortality compared to normoxemia. This result challenges our initial hypothesis. Our results were unaltered by a sensitivity analysis, where patients deceased within 24 h were excluded.

The beneficial effects of supplemental oxygen for the critically ill patient have remained undisputed for decades and have resulted in international guidelines on initial trauma management recommending high fractions of inspired oxygen. However, although it is suspected that hyperoxemia may be deleterious (due to increased oxidative stress, vasoconstriction, and potential hyperoxemic lung injury) [31], the evidence both in favor and against supplemental oxygen, and thus the risk of hyperoxemia, is almost non-existent in trauma patients [19].

Supplemental oxygen seems to possess a potential to rescue threatened neurons after brain injury or in the ischemic penumbra [32, 33], and it is known to prolong the safe apnea time [34]. Nevertheless, numerous physiologic arguments exist against liberal administration of oxygen in critically ill patients. For example, excess oxygen has been associated with the formation of reactive oxygen species which are detoxified in the mitochondria by a variety of antioxidants. Furthermore, acute states such as shock induce an increased production of reactive oxygen species worsening the imbalance between pro-oxidants and antioxidants [6].

In recent years, the optimal targets of both SpO_2_ and PaO_2_ have therefore been challenged in acutely ill patients. A large meta-analysis showed increased rates of mortality for patients with oxygen saturation (SpO_2_) above 96% compared to 94–96% [11]. However, as the trial sequential analysis was driven primarily by a single large randomized trial [35], the authors were unable to exclude a small beneficial effect of liberal oxygen. Only one RCT on trauma patients was included, and here no effect of liberal oxygen was observed. Another meta-analysis on patients with cardiac arrest showed beneficial effects of oxygen intra-arrest while post-arrest arrest hyperoxemia was associated with increased mortality [36]. A recent systematic review found a higher all-cause mortality in ICU patient with hyperoxemia [25]; however, in subgroup analyses on patients with TBI and patients on mechanical ventilation, results were inconclusive.

In trauma patients, studies on liberal versus conservative oxygen approaches are sparse. To date, only two small RCTs have been done on patients with TBI, and here, one found difference between a liberal and restrictive oxygen approach on mortality [17], and the other found no differences in terms of neurological outcome [37]. Furthermore, the few retrospective studies available have shown inconsistent results: one recent large study showed no difference in in-hospital mortality between hyperoxemic and normoxemic trauma patients [18], others have shown a deleterious effect of hyperoxemia [38, 39], and yet two studies have found a strong relationship between hyperoxemia and better long-term, functional, and cognitive outcomes [24, 40]. As such the physiologic consequences of hyperoxemia on outcomes after TBI remain uncovered. In several studies, a decrease in cerebral perfusion of up to 30% has been observed in individuals exposed to hyperoxia [41–43], while other studies have suggested that hyperoxia aids in one of the cornerstones in treatment of traumatic brain injury: decreasing intracranial pressure [44–46]. Supplemental oxygen could also be beneficial in TBI by simply increasing the level of oxygen in the brain. In stroke patients, supplemental oxygen has been proposed to rescue threatened neurons, and thus the brain, from further deterioration [47]. Nonetheless, studies so far have failed to show an association between supplemental oxygen and improved physical function [35, 48]. Further research in larger cohorts should look into this to help uncover the induced pathways.

In accordance with several of the above studies, we found a clinical benefit of early hyperoxemia in the current study. Of note, however, all the latter studies focus solely on trauma patients with TBI, whereas we chose to include all trauma patients to present a broader and more pragmatic perspective, as isolated TBI may not always be evident in the acute phase. Nonetheless, in our subgroup analysis of patients with GCS < 8, our results were unchanged.

The comparison of studies on hyperoxemia is difficult as some studies compare SpO_2_ values, others FiO_2_ values, and others PaO_2_ values. Besides, when utilizing the PaO_2,_ there is no consensus on the arbitrarily predetermined PaO_2_ cut-off [25]. In the current study, we chose to use 150 mmHg as the threshold for hyperoxemia as it presented a large percentage of our population (43%), and in addition, this approach has been used previously [22–25]. Numerous other studies have chosen values above 300 mmHg to present hyperoxemia, thereby considering values below 300 mmHg as normoxemic, which appears problematic. Furthermore, many studies have used the worst PaO_2_ (the highest PaO_2_) as their exposure variable [49, 50]. We chose to use the first PaO_2_ recorded at hospital admission to reflect the pre-hospital treatment. This has previously been done [38, 51]. Finally, the exposure duration should also be taken into account. As such, the attempt to answer whether or not hyperoxemia is harmful—in any patient population—should always aim to consider the variable measured (SpO_2_, PaO_2_ or FiO_2_), the concentration of the given variable, and the exposure duration.

Our results reflect the liberal use of pre-hospital oxygen administration of severe trauma patients, and we found a high percentage (43%) of patients with hyperoxemia at hospital admission. Although the duration of hyperoxemia in our study must be assumed to be relatively short (mean prehospital time from trauma until admission of 70 min), several studies have found that deleterious effects of hyperoxemia may occur already during the first hours of administration. For example, both human and animal data have shown development of lung injury after just a few hours of exposure to hyperoxemia [10, 22, 52]. Furthermore, prehospital supplemental oxygen administration for patients with myocardial infarction has been associated with increased myocardial injury and infarct size at 6 months [53], and in a recent study, an association between an even shorter exposure time to hyperoxia and mortality was found in mechanically ventilated patients in the emergency department [14].

Nevertheless, in a recent small single center observational study, authors found no impact on 30-day mortality in trauma patients with early hyperoxemia [54], and in our current study on trauma patients, we even found a significant association between hyperoxemia on admission and decreased mortality compared to normoxemia on admission. The threshold for potentially toxic concentrations and duration of administration of oxygen are poorly defined, and the mechanisms behind a favorable effect may, at least partly, be explained by hemodynamic stabilization during shock, improvement in tissue bed oxygenation in both peri-contusional and remote neuronal tissue, and more aerobic neural metabolic profiles [55]. These could be some of the explanation behind a positive effect of short-term hyperoxemia in the current study along with the actual ability of the affected individual to increase their PaO_2_ as demonstrated in the PaO_2_/FiO_2_ sensitivity analysis. Regarding the exact threshold, the current study also shows that this may point towards mild hyperoxemia being the most beneficial, as the beneficial effect seemed to decrease when a higher PaO_2_ was used to define hyperoxemia.

### Limitations

The primary limitation of the current study lies within its retrospective design, where, for instance, missing data often is seen. In our study, in-hospital mortality was unfortunately missing in 18%. Furthermore, the PaO_2_ value was also missing in a substantial proportion of patients, leaving these patients for exclusion. It is impossible to know whether these were missing completely at random or not. However, for a large proportion, they seem to be missing completely at random, as other results of an arterial blood gas were available. Nonetheless, the large number of included patients allowed not only the propensity score analysis to include all the necessary variables for corrections but also important subgroup analyses. One must, however, keep in mind that the risk of hidden confounders still exists. Furthermore, although the first PaO_2_ recorded at hospital admission partly represents the prehospital management, the median of several consecutive PaO_2_’s may have provided a more accurate picture. Moreover, in contrast to some other retrospective studies, we chose not to include a comparison group of hypoxemic patients, as the deleterious effect of hypoxemia is well established. This allows a cleaner comparison to the randomized trials available, where randomization is aimed at normoxemia versus hyperoxemia, thus not including a hypoxemic group.

We chose in-hospital mortality as our primary outcome as this seemed to be the most patient centered outcome available in the database. However, in future studies, other outcomes such as lactate levels and catecholamine administration could be interesting to look at, to gain a deeper understanding of the resulting physiological changes with different PaO_2_ levels.

Finally, the results of this study are based upon the French pre-hospital system which is characterized by the presence of emergency physicians in the field. The characteristics of the patients and the nature of their initial management can therefore not easily be extrapolated to EMS systems in other countries. Our results must therefore be compared with other systems of prehospital care.

## Conclusion

In the current study, we found early hyperoxemia in severe trauma patients to be associated with a reduced in-hospital mortality. This result may support systematic administration of oxygen in trauma patients during the initial management in the prehospital setting, but the retrospective nature of the study warrants its careful interpretation. The study calls for a randomized clinical trial to further investigate this association.

## Supplementary information

**Additional file 1.** Supplementary table on baseline characteristics for hypoxemic patients (PaO_2_ < 60 mmHg).

**Additional file 2.** Supplementary table on baseline characteristics for intubated vs spontaneously breathing patients.

**Additional file 3.** Multivariate full model including factors used in propensity score.

## Data Availability

The datasets generated during and/or analyzed during the current study may be available from the corresponding author on reasonable request.

## Abbreviations

ASA score: American Society of Anesthesiologists score
ED: Emergency department
FiO_2_: High inspired concentrations of oxygen
GCS: Glasgow Coma Scale
ICU: Intensive care unit
IPTW: Inverse probability of treatment weighting
IQR: Interquartile ranges
PaO_2_: Arterial oxygen partial pressure
RCT: Randomized controlled trials
SD: Standard deviations
SMUR: *Service Mobile d’Urgence et de Réanimation*
SpO_2_: Oxygen saturation
TBI: Traumatic brain injury

## References

[1] WHO | Injuries and violence: the facts. Available from: http://www.who.int/violence_injury_prevention/key_facts/en/. [cited 2017 Aug 11].

[2] Cost of Injury & Calculators | WISQARS | Injury Center | CDC. 2018. Available from: https://www.cdc.gov/injury/wisqars/cost/index.html. [cited 2019 May 10].

[3] American College of Surgeons. ATLS: Advanced Trauma Life Support for Doctors (Student Course Manual), 9th edition. 2012.

[4] Mosby. PHTLS: Basic and Advanced Prehospital Trauma Life Support. 5 edn. 2003.

[5] Cornet AD, Kooter AJ, Peters MJ, Smulders YM (2013). The potential harm of oxygen therapy in medical emergencies. Crit Care.

[6] Damiani E, Donati A, Girardis M (2018). Oxygen in the critically ill: friend or foe?. Curr Opin Anaesthesiol.

[7] Nagato AC, Bezerra FS, Lanzetti M, Lopes AA, Silva MAS, Porto LC (2012). Time course of inflammation, oxidative stress and tissue damage induced by hyperoxia in mouse lungs. Int J Exp Pathol.

[8] Schwingshackl A, Lopez B, Teng B, Luellen C, Lesage F, Belperio J (2017). Hyperoxia treatment of TREK-1/TREK-2/TRAAK-deficient mice is associated with a reduction in surfactant proteins. Am J Physiol Lung Cell Mol Physiol.

[9] Aboab J, Jonson B, Kouatchet A, Taille S, Niklason L, Brochard L (2006). Effect of inspired oxygen fraction on alveolar derecruitment in acute respiratory distress syndrome. Intensive Care Med.

[10] Staehr-Rye AK, Meyhoff CS, Scheffenbichler FT, Vidal Melo MF, Gätke MR, Walsh JL (2017). High intraoperative inspiratory oxygen fraction and risk of major respiratory complications. BJA Br J Anaesth.

[11] Chu DK, Kim LH-Y, Young PJ, Zamiri N, Almenawer SA, Jaeschke R (2018). Mortality and morbidity in acutely ill adults treated with liberal versus conservative oxygen therapy (IOTA): a systematic review and meta-analysis. Lancet Lond Engl.

[12] You J, Fan X, Bi X, Xian Y, Xie D, Fan M (2018). Association between arterial hyperoxia and mortality in critically ill patients: a systematic review and meta-analysis. J Crit Care.

[13] Helmerhorst HJ, Schultz MJ, van der Voort PH, Bosman RJ, Juffermans NP, de Jonge E (2014). Self-reported attitudes versus actual practice of oxygen therapy by ICU physicians and nurses. Ann Intensive Care.

[14] Page D, Ablordeppey E, Wessman BT, Mohr NM, Trzeciak S, Kollef MH (2018). Emergency department hyperoxia is associated with increased mortality in mechanically ventilated patients: a cohort study. Crit Care Lond Engl.

[15] Suzuki S, Eastwood GM, Peck L, Glassford NJ, Bellomo R (2013). Current oxygen management in mechanically ventilated patients: a prospective observational cohort study. J Crit Care.

[16] Helmerhorst HJF, Schultz MJ, van der Voort PHJ, Bosman RJ, Juffermans NP, de Wilde RBP (2016). Effectiveness and clinical outcomes of a two-step implementation of conservative oxygenation targets in critically ill patients: a before and after trial. Crit Care Med.

[17] Taher A, Pilehvari Z, Poorolajal J, Aghajanloo M (2016). Effects of normobaric hyperoxia in traumatic brain injury: a randomized controlled clinical trial. Trauma Mon.

[18] Ó Briain D, Nickson C, Pilcher DV, Udy AA (2018). Early hyperoxia in patients with traumatic brain injury admitted to intensive care in Australia and New Zealand: a retrospective multicenter cohort study. Neurocrit Care.

[19] Eskesen TG, Baekgaard JS, Steinmetz J, Rasmussen LS (2018). Initial use of supplementary oxygen for trauma patients: a systematic review. BMJ Open.

[20] von Elm E, Altman DG, Egger M, Pocock SJ, Gøtzsche PC, Vandenbroucke JP (2014). The Strengthening the Reporting of Observational Studies in Epidemiology (STROBE) statement: guidelines for reporting observational studies. Int J Surg Lond Engl.

[21] Hamada SR, Gauss T, Duchateau F-X, Truchot J, Harrois A, Raux M (2014). Evaluation of the performance of French physician-staffed emergency medical service in the triage of major trauma patients. J Trauma Acute Care Surg.

[22] Girardis M, Busani S, Damiani E, Donati A, Rinaldi L, Marudi A (2016). Effect of conservative vs conventional oxygen therapy on mortality among patients in an intensive care unit: the oxygen-ICU randomized clinical trial. JAMA..

[23] Jouffroy R, Saade A, Saint Martin LC, Philippe P, Carli P, Vivien B (2019). Prognosis value of partial arterial oxygen pressure in patients with septic shock subjected to pre-hospital invasive ventilation. Am J Emerg Med.

[24] Alali AS, Temkin N, Vavilala MS, et al. Matching early arterial oxygenation to long-term outcome in severe traumatic brain injury: target values. J Neurosurg. 2019;132(2):537–44. 10.3171/2018.10.JNS18964. 10.3171/2018.10.JNS18964 30738409

[25] Ni Y-N, Wang Y-M, Liang B-M, Liang Z-A (2019). The effect of hyperoxia on mortality in critically ill patients: a systematic review and meta analysis. BMC Pulm Med.

[26] White IR, Royston P, Wood AM (2011). Multiple imputation using chained equations: issues and guidance for practice. Stat Med.

[27] Mitra R, Reiter JP (2016). A comparison of two methods of estimating propensity scores after multiple imputation. Stat Methods Med Res.

[28] Austin PC (2011). An introduction to propensity score methods for reducing the effects of confounding in observational studies. Multivar Behav Res.

[29] Definition Task Force ARDS, Ranieri VM, Rubenfeld GD, Thompson BT, Ferguson ND, Caldwell E (2012). Acute respiratory distress syndrome: the Berlin Definition. JAMA..

[30] R Foundation for Statistical Computing, Vienna, Austria. R Core Team (2016). R: A language and environment for statistical computing.

[31] Asfar P, Singer M, Radermacher P (2015). Understanding the benefits and harms of oxygen therapy. Intensive Care Med.

[32] Pountain SJ, Roffe C (2012). Does routine oxygen supplementation in patients with acute stroke improve outcome?. BMJ..

[33] Michalski D, Härtig W, Schneider D, Hobohm C (2011). Use of normobaric and hyperbaric oxygen in acute focal cerebral ischemia - a preclinical and clinical review. Acta Neurol Scand.

[34] Kelly C (2014). Oxygen therapy: time to move on?. Ther Adv Respir Dis.

[35] Roffe C, Nevatte T, Sim J, Bishop J, Ives N, Ferdinand P (2017). Effect of routine low-dose oxygen supplementation on death and disability in adults with acute stroke: the stroke oxygen study randomized clinical trial. JAMA..

[36] Patel JK, Kataya A, Parikh PB (2018). Association between intra- and post-arrest hyperoxia on mortality in adults with cardiac arrest: a systematic review and meta-analysis. Resuscitation..

[37] Lång M, Skrifvars MB, Siironen J, Tanskanen P, Ala-Peijari M, Koivisto T (2018). A pilot study of hyperoxemia on neurological injury, inflammation and oxidative stress. Acta Anaesthesiol Scand.

[38] Davis DP, Meade W, Sise MJ, Kennedy F, Simon F, Tominaga G (2009). Both hypoxemia and extreme hyperoxemia may be detrimental in patients with severe traumatic brain injury. J Neurotrauma.

[39] Rincon F, Kang J, Vibbert M, Urtecho J, Athar MK, Jallo J (2014). Significance of arterial hyperoxia and relationship with case fatality in traumatic brain injury: a multicentre cohort study. J Neurol Neurosurg Psychiatry.

[40] Asher SR, Curry P, Sharma D, Wang J, O’Keefe GE, Daniel-Johnson J (2013). Survival advantage and PaO2 threshold in severe traumatic brain injury. J Neurosurg Anesthesiol.

[41] Watson NA, Beards SC, Altaf N, Kassner A, Jackson A (2000). The effect of hyperoxia on cerebral blood flow: a study in healthy volunteers using magnetic resonance phase-contrast angiography. Eur J Anaesthesiol.

[42] Bulte DP, Chiarelli PA, Wise RG, Jezzard P (2007). Cerebral perfusion response to hyperoxia. J Cereb Blood Flow Metab.

[43] Borzage MT, Bush AM, Choi S, Nederveen AJ, Václavů L, Coates TD (2016). Predictors of cerebral blood flow in patients with and without anemia. J Appl Physiol (1985).

[44] Tolias CM, Reinert M, Seiler R, Gilman C, Scharf A, Bullock MR (2004). Normobaric hyperoxia--induced improvement in cerebral metabolism and reduction in intracranial pressure in patients with severe head injury: a prospective historical cohort-matched study. J Neurosurg.

[45] Reinert M, Barth A, Rothen HU, Schaller B, Takala J, Seiler RW (2003). Effects of cerebral perfusion pressure and increased fraction of inspired oxygen on brain tissue oxygen, lactate and glucose in patients with severe head injury. Acta Neurochir (Wien).

[46] Rockswold SB, Rockswold GL, Zaun DA, Liu J (2013). A prospective, randomized phase II clinical trial to evaluate the effect of combined hyperbaric and normobaric hyperoxia on cerebral metabolism, intracranial pressure, oxygen toxicity, and clinical outcome in severe traumatic brain injury. J Neurosurg.

[47] Singhal AB (2006). Oxygen therapy in stroke: past, present, and future. Int J Stroke.

[48] Rønning OM, Guldvog B (1999). Should stroke victims routinely receive supplemental oxygen? A quasi-randomized controlled trial. Stroke..

[49] Helmerhorst HJF, Roos-Blom M-J, van Westerloo DJ, de Jonge E (2015). Association between arterial hyperoxia and outcome in subsets of critical illness: a systematic review, meta-analysis, and meta-regression of cohort studies. Crit Care Med.

[50] Helmerhorst HJF, Arts DL, Schultz MJ, van der Voort PHJ, Abu-Hanna A, de Jonge E (2017). Metrics of arterial hyperoxia and associated outcomes in critical care. Crit Care Med.

[51] Kilgannon JH, Jones AE, Parrillo JE, Dellinger RP, Milcarek B, Hunter K (2011). Relationship between supranormal oxygen tension and outcome after resuscitation from cardiac arrest. Circulation..

[52] Andrade PV, dos Santos JM, Silva HCA, Wilbert DD, Cavassani SS, Oliveira-Júnior IS (2014). Influence of hyperoxia and mechanical ventilation in lung inflammation and diaphragm function in aged versus adult rats. Inflammation..

[53] Dion S, Karen S, Stephen B, Ziad N, Michael S, Bray Janet E (2015). Air versus oxygen in ST-segment–elevation myocardial infarction. Circulation..

[54] Harpsø M, Granfeldt A, Løfgren B, Deakin CD (2019). No effect of hyperoxia on outcome following major trauma. Open Access Emerg Med.

[55] Bitterman H (2009). Bench-to-bedside review: oxygen as a drug. Crit Care Lond Engl..

