# Supplementary material for: Early hyperoxemia is associated with lower adjusted mortality after severe trauma: results from a French registry
Source: Crit Care. 2020 Oct 12;24:604. doi: 10.1186/s13054-020-03274-x (PMC7549241; doi:10.1186/s13054-020-03274-x)
Supplement: Supplementary file 1 — Additional file 1. Supplementary table on baseline characteristics for hypoxemic patients (PaO2 < 60 mmHg). [file 13054_2020_3274_MOESM1_ESM.docx]

| **Additional file 1 - Baseline characteristics of hypox~~a~~emic patients (n= 428)** | |
| --- | --- |
| *Results are presented as medians with [interquartile ranges], numbers with (percentages), or as otherwise indicated* | |
| Age, mean (sd) | 41 (19) |
| Sex (female) | 83 (19.5) |
| ASA-score> 1 | 144 (35.5) |
| Mechanism of injury |  |
| *Falls from height* | 87 (20.4) |
| *Falls from standing* | 13 (3.0) |
| *Vehicle incident/collision* | 237 (55.5) |
| *Shootings* | 66 (15.5) |
| *Fight* | 7 (1.6) |
| *Other* | 17 (4.0) |
| Site of injury |  |
| *Head and Neck* | 170 (44.2) |
| *Face* | 87 (22.6) |
| *Abdomen* | 105 (27.3) |
| *Chest* | 190 (49.4) |
| *External* | 85 (22.1) |
| *Extremities* | 218 (56.6) |
| Duration of prehospital care (minutes), median [IQR] | 66 [45-90] |
| Prehospital systolic blood pressure (mmHg) | 128 [110, 144] |
| Prehospital heart rate (bpm) | 90 [ 77, 107] |
| Prehospital intubation | 97 (22.9) |
| Prehospital GCS score | 15 [14, 15] |
| Values on hospital arrival |  |
| pH | 7.3 [7.3, 7.4] |
| PaO_2_ | 43 [33, 52] |
| PCO_2_ | 46 [39, 57] |
| Temperature (°C) | 36.6 [35.9, 37.1] |
| Lactate (mmol/L) | 2.2 [1.5, 3.5] |
| Creatinine (µmol/L) | 82 [69, 98] |
| Hemoglobin (mmol/L) | 13.5 [11.9, 14.6] |
| Catecholamine administration | 56 (13.6) |
| Fluid replacement | 500 [250, 1,000] |
| ISS score | 11 [ 5, 20] |
| ISS score> 15 | 149 (38.3) |
| Traumatic Brain Injury | 94 (22.6) |
| Hemorrhagic shock | 43 (10.3) |
| In-hospital mortality | 39 (9.1) |
|  |  |

*Abbreviations: ASA, American Society of Anesthesiologists; GCS, Glasgow Coma Scale score; ISS, Injury Severity Score; Hemorrhagic Shock (defined as administration of at least four units of packed red blood cells within six hours); Fluid replacement (mL of colloids and/or crystalloids)*

*The provided pre-hospital vital signs are the first vital signs recorded on-scene.*
