# Supplementary material for: Early hyperoxemia is associated with lower adjusted mortality after severe trauma: results from a French registry
Source: Crit Care. 2020 Oct 12;24:604. doi: 10.1186/s13054-020-03274-x (PMC7549241; doi:10.1186/s13054-020-03274-x)
Supplement: Supplementary file 2 — Additional file 2. Supplementary table on baseline characteristics for intubated vs spontaneously breathing patients. [file 13054_2020_3274_MOESM2_ESM.docx]

| **Additional file 2** - Baseline Characteristics of spontaneously breathing patients and patients on mechanical ventilation | | | |  |
| --- | --- | --- | --- | --- |
| *Results are presented as medians with [interquartile ranges], numbers with (percentages), or as otherwise indicated* | | | |  |
|  | Spontaneously breathing (n=3967) | Mechanical ventilation (n=1840) | p-value |  |
| Age, mean (sd) | 41.9 (18.3) | 41.8 (18.3) | 0.82 |  |
| Sex (female) | 854 (21.6) | 395 (21.6) | 0.92 |  |
| Prehospital GCS score | 15.0 [15.0, 15.0] | 7.0 [4.0, 13.0] | < 1e-04 |  |
| Prehospital systolic blood pressure (mmHg) | 128.0 [110.0, 140.0] | 125.0 [106.0, 145.0] | 0.049 |  |
| Prehospital heart rate (bpm) | 88.0 [75.0, 101.0] | 90.0 [75.0, 110.0] | < 1e-04 |  |
| Values on hospital arrival |  |  |  |  |
| pH | 7.4 [7.3, 7.4] | 7.3 [7.2, 7.4] | < 1e-04 |  |
| PaO_2_ | 116.0 [90.0, 180.0] | 193.0 [121.0, 286.2] | < 1e-04 |  |
| PCO_2_ | 39.0 [35.0, 43.0] | 42.0 [37.0, 48.0] | < 1e-04 |  |
| Temperature (°C) | 36.6 [36.1, 37.1] | 36.0 [35.2, 36.8] | < 1e-04 |  |
| Lactate (mmol/L) | 1.8 [1.2, 2.7] | 2.3 [1.4, 3.6] | < 1e-04 |  |
| Creatinine (µmol/L) | 76.0 [65.0, 90.0] | 78.0 [64.0, 97.0] | 0.011 |  |
| Hemoglobin (mmol/L) | 13.3 [11.9, 14.4] | 12.4 [10.7, 13.8] | < 1e-04 |  |
| Catecholamine administration | 207 (5.3) | 602 (33.7) | < 1e-04 |  |
| Fluid replacement | 500.0 [250.0, 850.0] | 1000.0 [500.0, 1500.0] | < 1e-04 |  |
| Hemorrhagic shock | 241 (6.2) | 295 (16.3) | < 1e-04 |  |
| Traumatic Brain Injury | 652 (16.7) | 1145 (63.1) | < 1e-04 |  |
| ISS score | 13.0 [8.0, 21.0] | 25.0 [16.0, 33.0] | < 1e-04 |  |
| In-hospital mortality | 82 (2.4) | 358 (21.4) | < 1e-04 |  |
|  |  |  |  |  |
| *GCS, Glasgow Coma Scale score; Fluid replacement (mL of colloids and/or crystalloids); Hemorrhagic Shock (defined as administration of at least four units of packed red blood cells within six hours); ISS, Injury Severity Score* | | | | |
|  |  |  |  |  |
|  |  |  |  |  |
|  |  |  |  |  |
