# Supplementary material for: Early hyperoxemia is associated with lower adjusted mortality after severe trauma: results from a French registry
Source: Crit Care. 2020 Oct 12;24:604. doi: 10.1186/s13054-020-03274-x (PMC7549241; doi:10.1186/s13054-020-03274-x)
Supplement: Supplementary file 3 — Additional file 3. Multivariate full model including factors used in propensity score. [file 13054_2020_3274_MOESM3_ESM.docx]

Additional file 3

*Multivariate full model including factors used in propensity score*

| Variable | Univariate | | Multivariate | |
| --- | --- | --- | --- | --- |
|  | OR [95%CI] | p | OR [95%CI] | p |
| PAO_2_>150 | 1.37 [1.14-1.66] | 0.001 | 0.71 [0.54-0.92] | 0.011 |
| Age | 1.03 [1.03-1.04] | <0.0001 | 1.03 [1.02-1.04] | <0.0001 |
| Sex (Male) | 0.77 [0.62-0.95] | 0.019 | 1.15 [0.84-1.57] | 0.39 |
| Initial GCS score | 0.76 [0.74-0.78] | <0.0001 | 0.83 [0.8-0.86] | <0.0001 |
| ASA-score > 1 | 2.33 [1.91-2.86] | <0.0001 | 1.15 [0.82-1.6] | 0.42 |
| Systolic blood pressure | 0.99 [0.98-0.99] | <0.0001 | 1 [1-1] | 0.61 |
| Heart rate | 0.99 [0.98-0.99] | 0.005 | 0.99 [0.99-1] | 0.002 |
| Prehospital intubation | 8.85 [7.07-11.1] | <0.0001 | 1.23 [0.86-1.77] | 0.26 |
| Temperature | 0.46 [0.42-0.5] | <0.0001 | 0.5 [0.37-0.66] | <0.0001 |
| Hemoglobin | 0.73 [0.7-0.76] | <0.0001 | 0.88 [0.83-0.93] | <0.0001 |
| Traumatic Brain Injury | 5.65 [4.6-6.92] | <0.0001 | 1.74 [1.28-2.36] | 0.0004 |
| ISS score> 15 | 10.22 [7.42-14.07] | <0.0001 | 3.35 [2.26-4.98] | <0.0001 |
| Creatinine | 1.01 [1.01-1.01] | <0.0001 | 1.01 [1-1.01] | <0.0001 |
| Catecholamine administration | 7.81 [6.35-9.62] | <0.0001 | 1.88 [1.4-2.54] | <0.0001 |
| Lactate | 1.33 [1.29-1.37] | <0.0001 | 1.22 [1.17-1.27] | <0.0001 |
| Fluid replacement | 1 [1-1] | <0.0001 | 1 [1-1] | 0.11 |
